# Novel anatomically contoured locking plate shows improved anatomic conformity vs. a conventional T-shaped plate in anterior popliteus transtibial-tuberosity high tibial osteotomy: a radiologic comparison

**DOI:** 10.3389/fsurg.2025.1722297

**Published:** 2025-11-25

**Authors:** Zhonghui Yao, Jialin He, Qiuhan Chen, Zihao Zou, Da Lei, Zhanyu Wu, Chuan Ye

**Affiliations:** 1 Department of Orthopaedics, The Affiliated Hospital of Guizhou Medical University, Guiyang, China; 2 Center for Tissue Engineering and Stem Cell Research, Guizhou Medical University, Guiyang, China; 3 Department of Orthopaedics, The Affiliated Hospital of Zunyi Medical University, Zunyi, China

**Keywords:** high tibial osteotomy, plate–bone gap, screw trajectory, anterior popliteus transtibial-tuberosity high tibial osteotomy, T-shaped locking plate, computed tomography, retrospective study

## Abstract

**Background:**

High tibial osteotomy (HTO) corrects varus malalignment and unloads the medial knee compartment, yet the conventional T-shaped locking plate may cause discomfort due to suboptimal anatomic conformity. We developed a novel locking plate to improve anatomic fit and fixation stability and compared it with the T-shaped plate in Anterior Popliteus Transtibial-tuberosity high tibial osteotomy (APTT-HTO).

**Methods:**

This retrospective comparative study included 14 consecutive patients who underwent APTT-HTO between August 2024 and June 2025. Patients were grouped by implant type (novel plate: *n* = 7; T-shaped plate: *n* = 7). Postoperative CT quantified plate orientation/position, plate–bone conformity (gap and position mismatch), screw alignment, and standard alignment parameters [hip–knee–ankle angle [HKA], posterior tibial slope [PTS]].

**Results:**

The novel plate showed more posteromedial placement and superior anatomic conformity. The plate angle relative to the posterior tibial condylar reference line was larger with the novel plate (74.49° ± 8.76°) than with the T-shaped plate (62.62° ± 7.05°, *P* < 0.05). The proximal central screw–plate angle was smaller (5.70° ± 4.80° vs. 27.48° ± 6.05°, *P* < 0.05), the plate–bone gap was reduced (1.47 (1.35–2.28) vs. 3.12 (2.70–3.26) mm, *P* < 0.05), and plate position mismatch was lower (18.86 (14.32–23.87) vs. 34.31 (30.57–42.08) %, *P* < 0.05). Proximal and distal offsets considered separately were not significantly different (*P* > 0.05). Both groups achieved the planned coronal correction (*P* < 0.05), and the sagittal PTS remained unchanged (*P* < 0.05).

**Conclusions:**

In this CT-based cohort, the novel anatomically contoured locking plate achieved superior anatomic conformity in APTT-HTO—characterized by more posteromedial positioning, a more favorable screw trajectory, and improved plate–bone apposition—compared with a conventional T-shaped plate. These findings warrant confirmation in larger, prospective studies.

## 1 Introduction

High tibial osteotomy (HTO) is an established procedure for medial unicompartmental knee osteoarthritis, relieving symptoms by realigning the lower-limb mechanical axis to offload the medial compartment (1). Compared with lateral closing-wedge HTO, medial opening-wedge HTO (OWHTO) has gained wider adoption in recent years because it allows controlled intraoperative correction and avoids fibular osteotomy, among other advantages (2). However, the mechanical stability of the osteotomy site after OWHTO remains a major concern; inadequate fixation can result in loss of correction and complications such as nonunion (3, 4). The T-shaped locking plate, a T-shaped locking compression fixation system, has—owing to its favourable biomechanical properties—significantly reduced the incidence of delayed union and nonunion after OWHTO (5). Nevertheless, commonly used fixation plates still have limitations in implant positioning and anatomical conformity. Evidence indicates that both plate placement and the orientation of the proximal screws are critical determinants of fixation stability in OWHTO (6). Positioning the plate on the posteromedial tibia provides greater stability than anteromedial placement (4). Orienting the proximal screws toward the lateral hinge-rather than the posterior cortex-improves fixation stability and reduces the risk of injury to posterior neurovascular structures (7). In addition, a plate geometry that conforms to the post-osteotomy medial tibial contour reduces the plate–bone gap, thereby enhancing construct strength (8).

In OWHTO, a biplanar osteotomy is commonly used to avoid patellar tendon injury by preserving an intact tibial tubercle and protecting its insertion. Our team previously proposed the Anterior Popliteus Transtibial-tuberosity high tibial osteotomy (APTT-HTO), which employs a single-plane osteotomy. Based on prior imaging and anatomical studies, the tibial tubercle can be divided proximodistally into A, B, C, and D zones; when the osteotomy line passes through zones B–D, the stability of the patellar tendon attachment is not appreciably compromised (9). Biomechanical studies and preliminary clinical application indicate that, while ensuring procedural safety, this technique optimisations the osteotomy-line design, streamlines intraoperative workflow, and effectively avoids patellar tendon injury and postoperative patella baja. Our team originally designed a novel anatomically contoured locking plate, motivated mainly by the need to overcome the shortcomings of the conventional T-shaped locking plate. It is an anatomically contoured locking plate whose geometry conforms more closely to the proximal medial tibia, allowing a tighter apposition to the medial and posteromedial cortices at the osteotomy site and thereby reducing soft-tissue irritation; It effectively resists axial load and provides robust buttress support to the tibial plateau; distributes stress evenly to mitigate stress concentration and thereby prevent screw or plate breakage; orients screw trajectories to avoid major neurovascular structures; and effectively maintains the posterior tibial slope and wedge opening height, preventing loss of correction. We therefore hypothesised that, in APTT-HTO, the novel anatomically contoured locking plate would show a more rational implant position and screw trajectory than T-shaped locking plate on postoperative imaging, thereby enhancing fixation stability and facilitating bone healing.

**Figure 1:**
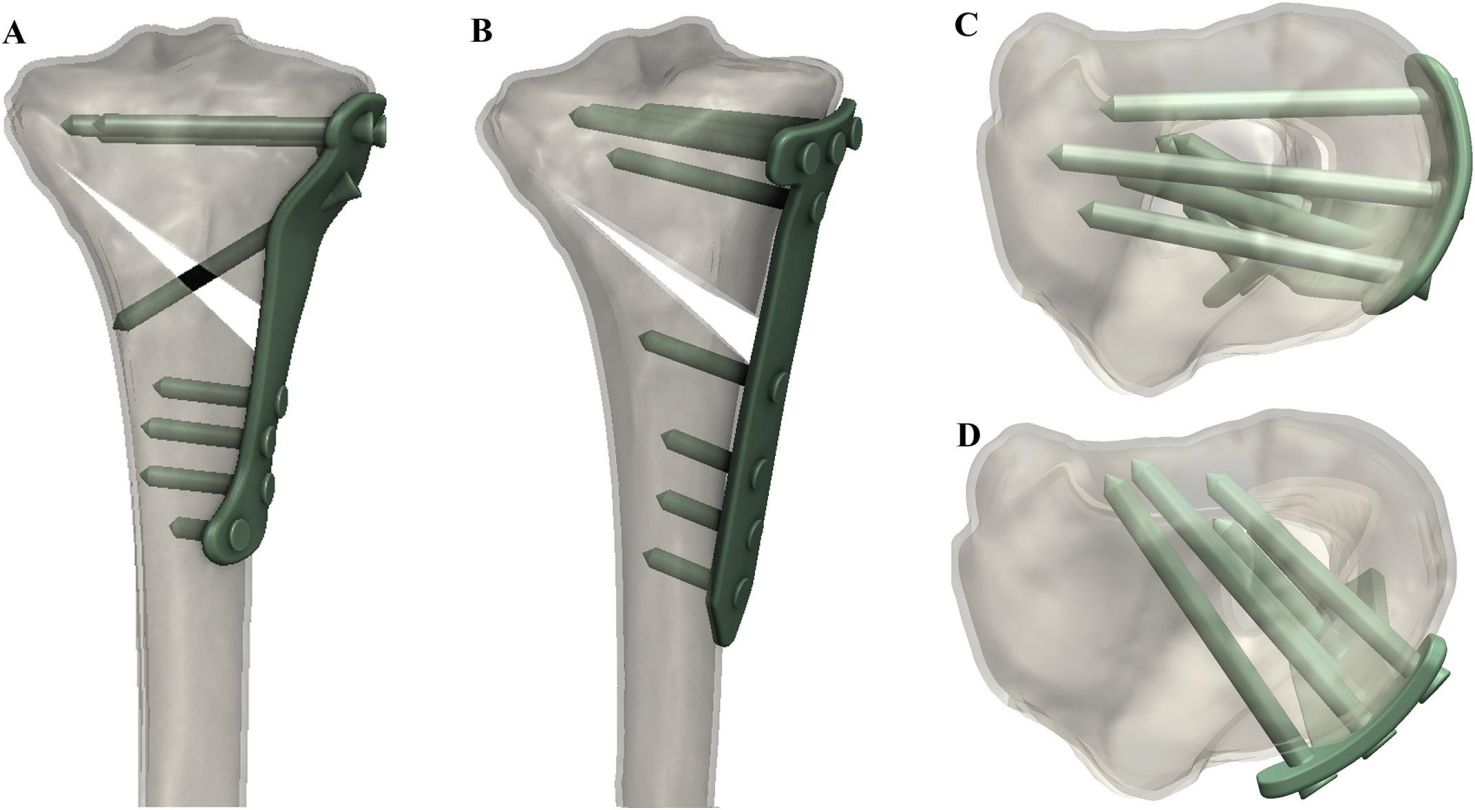
3D-reconstructed CT images showing **(A)** coronal view with the novel locking plate, **(B)** coronal view with the T-shaped locking plate, **(C)** axial view with the novel locking plate, and **(D)** axial view with the T-shaped locking plate.

This study measured and analysed postoperative imaging to compare the radiological characteristics of fixation between the novel locking plate and conventional T-shaped locking plate in APTT-HTO, aiming to verify the novel plate's advantages in implant positioning and anatomical matching, screw trajectory, and plate–bone apposition, thereby providing evidence for its clinical application (Figure 1).

## 2 Materials and methods

### 2.1 Study population

This retrospective study enrolled 14 patients with medial compartment knee osteoarthritis and varus deformity who underwent APTT-HTO at the Affiliated Hospital of Guizhou Medical University between August 2024 and June 2025. The protocol was approved by the Ethics Committee of Guizhou Medical University (approval No. 2025140k). Patients were allocated according to the fixation used into a T-shaped locking plate group and a novel locking plate group (*n* = 7 each). Baseline demographic and clinical characteristics are summarised in (Table 1).

**Table 1:** Demographic and preoperative data.

Variable	Novel locking plate	T-shaped locking plate
Age, year	66.1 ± 5.1	65.6 ± 6.2
Sex, M : F	1 : 6	0 : 7
Side, right : left	7 : 0	7 : 0
BMI, kg/m^2^	24.8 ± 1.9	25.8 ± 2.1
Preoperative mTFA (°)	6.54 ± 0.2	6.60 ± 0.54
Preoperative MPTA (°)	83.92 ± 2.59	86.48 ± 2.28
Preoperative tibial slope (°)	5.14 ± 1.99	5.43 ± 1.48
Preoperative HKA (°)	171.55 ± 2.82	175.20 ± 1.40

Values are presented as mean ± standard deviation. BMI, body mass index; HKA, hip-knee-ankle; MPTA, medial proximal tibial angle, mTFA, mechanical tibiofemoral angle.

#### 2.1.1 Inclusion and exclusion criteria

Inclusion criteria: (1) Primary degenerative osteoarthritis (non-inflammatory); (2) Plain radiographs showing isolated medial-compartment osteoarthritis with Kellgren–Lawrence grade III or IV, without severe patellofemoral osteoarthritis.

Exclusion criteria: (1) Advanced bicompartmental involvement beyond the medial compartment (inflammatory or degenerative); (2) Prior knee surgery or history of major knee trauma; rheumatoid arthritis or other inflammatory arthritides; periarticular tumour; or knee joint infection; (3) Severe cruciate ligament insufficiency; (4) Markedly limited ambulation or systemic conditions precluding surgery/anaesthesia; (5) Body mass index (BMI) > 32 kg/m^2^.

**Figure 2:**
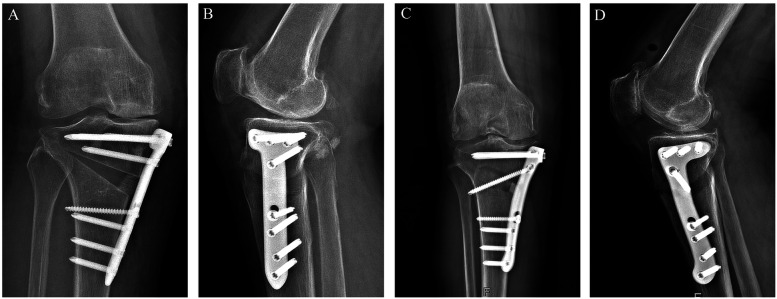
Features of the T-shaped locking plate and the novel locking plate. **(A)** Front view of the T-shaped locking plate; **(B)** Side (lateral) view of the T-shaped locking plate; **(C)** Front view of the novel locking plate; **(D)** Side (lateral) view of the novel locking plate.

Patients were stratified by implant into two groups: a T-shaped locking plate group (fixed with the conventional T-shaped locking plate) and a novel locking plate group, with 7 patients in each (Figure 2). We recorded demographic variables (age, sex, body mass index) and preoperative imaging parameters, including the HKA, MPTA, and PTS. All procedures were performed by the same orthopaedic surgical team.

Implant specifications: The novel locking plate is made of medical-grade titanium alloy (Ti-6Al-4V). The main dimensions are as follows: the T-shaped head has a slight curvature to match the medial contour of the proximal tibia. Overall length: 115 mm; plate thickness: 3 mm; shaft width: 16 mm. Proximal (T-head) holes (Ha–Hc): three threaded locking holes, compatible with 5.0-mm locking screws. Oblique osteotomy hole (Hd): a multi-axial locking hole designed to cross the osteotomy plane and draw the distal fragment toward the medial plate. Shaft holes (H1–H4): four threaded locking holes (for 5.0-mm locking screws). The distal edge adopts a curved transition design to better conform to the cortical curvature of the distal tibia and to reduce local prominence.

### 2.2 Surgical procedure

**Figure 3:**
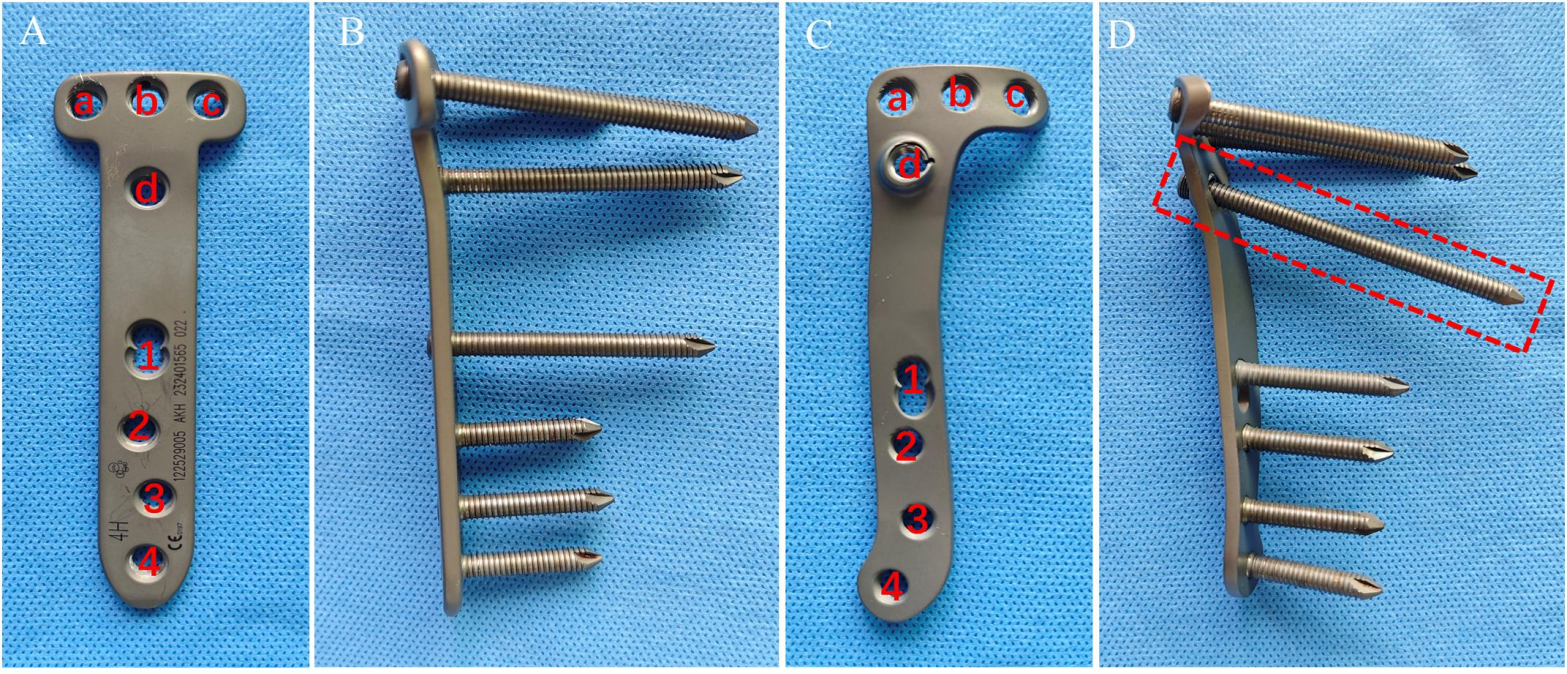
Anteroposterior (AP) and lateral views of each plate: **(A)** T-shaped locking plate-AP; **(B)** T-shaped locking plate-lateral; **(C)** novel locking plate-AP; **(D)** novel locking plate-lateral.

Through an anteromedial oblique incision at the proximal tibia, layers were dissected to expose the metaphysis. A Kirschner wire was inserted ∼1.5 cm distal to the superior margin of the pes anserinus, directed medially to superolaterally toward the proximal fibular head, and its position was confirmed under C-arm fluoroscopy. Using the K-wire as the reference line, a single-plane osteotomy of the proximal tibia was performed with an oscillating saw, preserving the lateral cortical hinge. After completion of the cut, the osteotomy gap was gradually opened with osteotomes; under fluoroscopic guidance the lower-limb mechanical axis was adjusted to the planned correction. An osteotomy spreader was then used to widen the gap to the predetermined height. A customized spacer block was inserted into the osteotomy gap to maintain alignment, the spreader was removed, and spacer stability was confirmed. The preselected plate was introduced through a minimally invasive corridor; after fluoroscopic confirmation of plate position, eight screws were placed sequentially to complete fixation. Final fluoroscopy verified the positions and lengths of the plate and screws. The only difference between groups was the type of plate implanted: the T-shaped locking plate in the T-shaped locking plate group vs. the novel locking plate in the novel plate group (Figure 3).

### 2.3 Postoperative management

Isometric quadriceps exercises and continuous passive motion (CPM) were initiated on postoperative day 1. Partial weight-bearing with crutches was allowed during the first 4 weeks after surgery. If tolerated, patients progressed to full weight-bearing from postoperative week 6.

### 2.4 Imaging assessment

All patients underwent full-length, non–weight-bearing bilateral lower-limb radiographs preoperatively and on postoperative day 2 to evaluate changes in the mechanical axis, and knee CT scans were obtained to assess implant positioning. In addition to routine alignment assessment, this study focused on postoperative CT analysis. Using the PACS software, the following five radiographic parameters were measured:

#### 2.4.1 Plate angle

**Figure 4:**
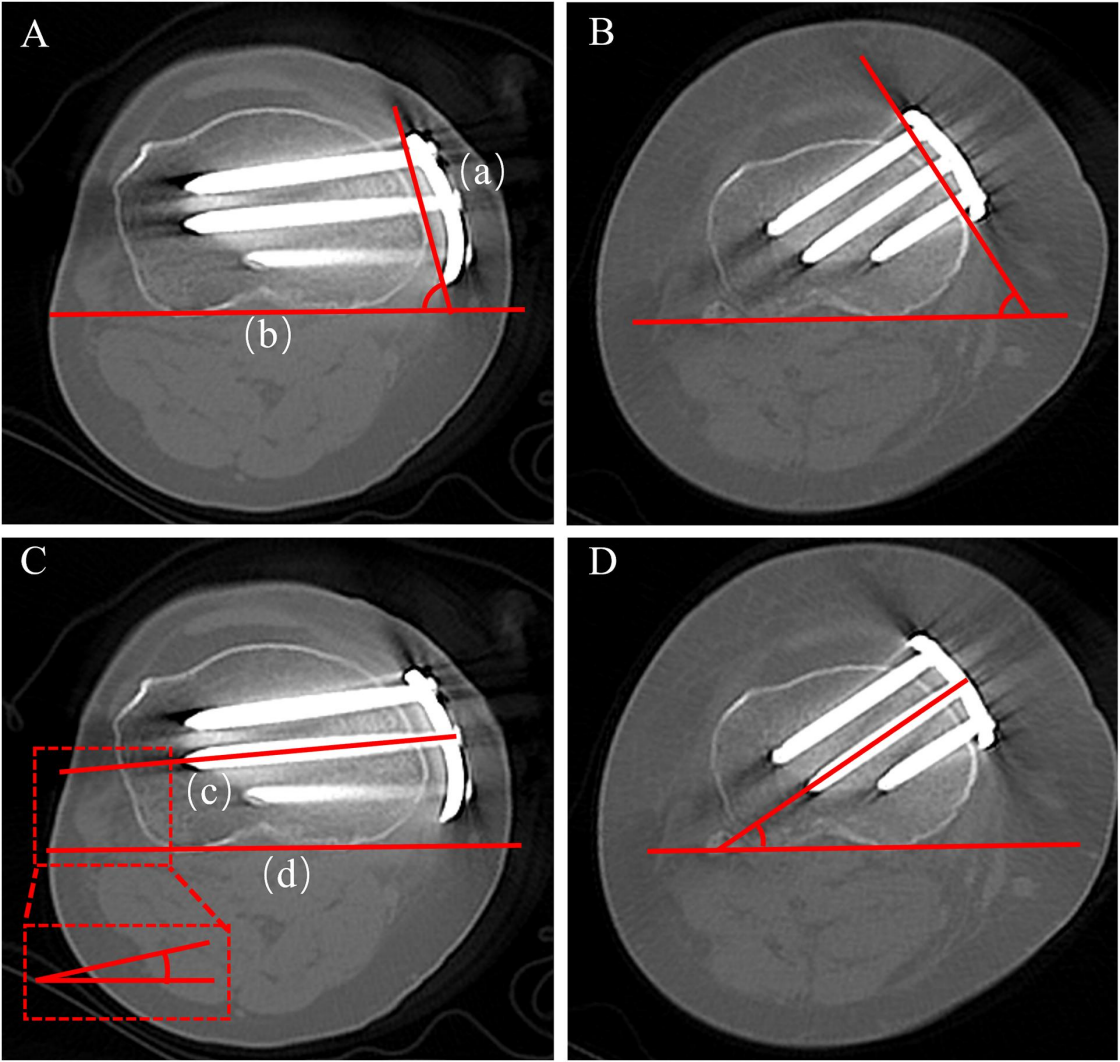
Measurement of plate angle and proximal central screw angle on axial CT. **(A)** Plate angle-novel locking plate; **(B)** plate angle-T-shaped locking plate; **(C)** proximal central screw angle-novel locking plate; **(D)** proximal central screw angle-T-shaped locking plate.

The inclination of the plate relative to the posterior tibial condylar reference line. On proximal axial CT images, draw line (a) connecting the anterior and posterior edges of the plate's proximal segment, and line (b) along the posterior tibial condylar reference line. The acute angle between lines (a) and (b) is defined as the plate angle; a larger value denotes a more posteromedial placement of the plate on the tibial axial plane (Figure 4).

#### 2.4.2 Screw angle and length

**Figure 5:**
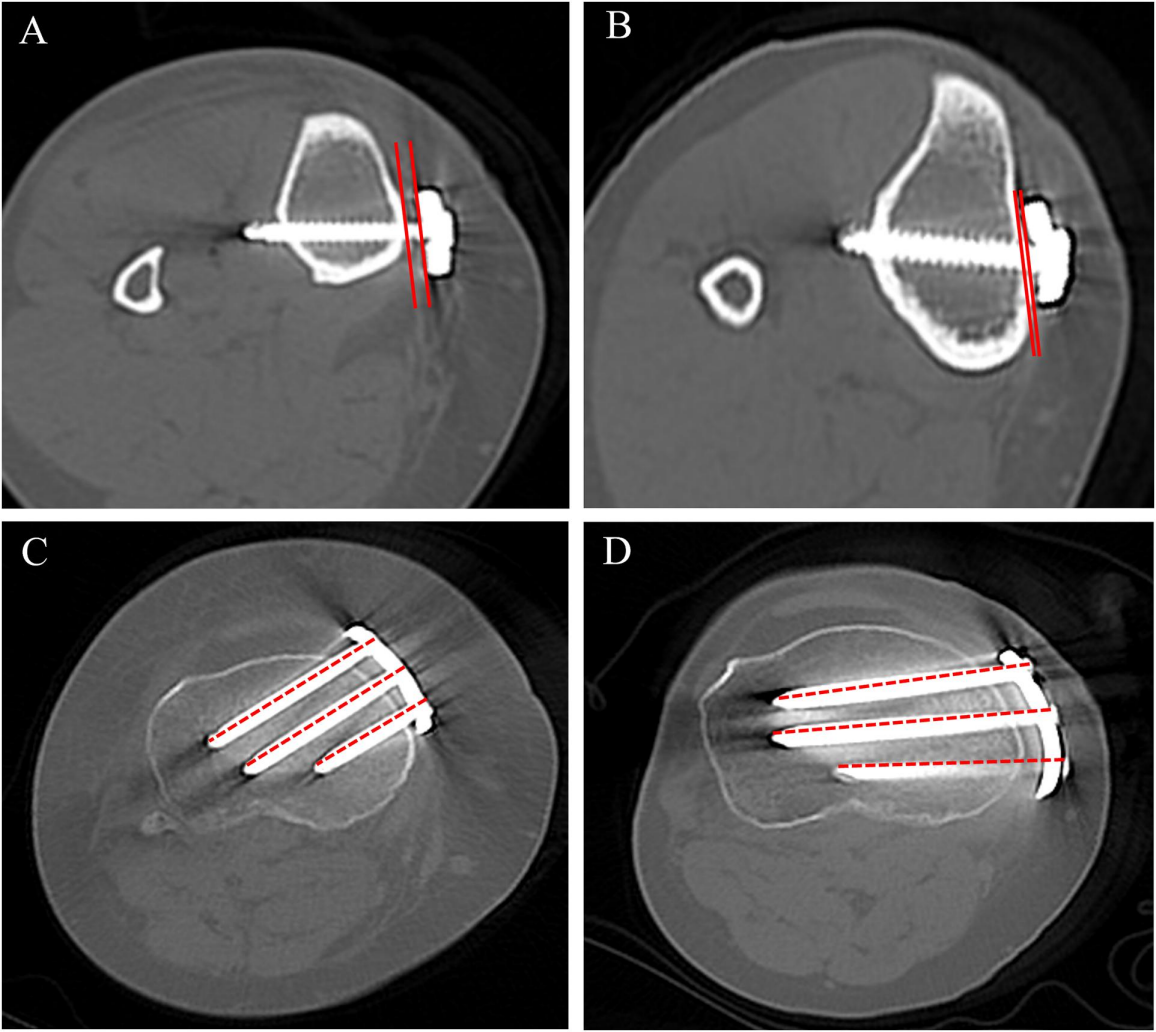
Axial CT measurements of (i) the vertical plate–bone gap (measured immediately inferior to the osteotomy) and (ii) the lengths of screws A, B, and C. **(A)** T-shaped locking plate-vertical plate–bone gap; **(B)** novel locking plate-vertical plate–bone gap; **(C)** T-shaped locking plate-lengths of screws A–C; **(D)** novel locking plate-lengths of screws A–C.

The inclination of the proximal central screw relative to the posterior tibial condylar reference line (Figure 4). On axial CT, measure the acute angle between line (c), the central axis of the proximal central screw, and line (d), the posterior tibial condylar reference line. A smaller angle indicates a trajectory directed toward the lateral hinge, whereas a larger angle indicates a trajectory toward the posterior cortex. On axial CT, the lengths of screws A, B, and C were measured separately (Figure 5).

#### 2.4.3 Plate–bone gap

The distance between the inner (bone-facing) surface of the plate and the tibial cortex at hole 1. On axial CT at the hole 1 level, draw a line along the inner surface of the plate and a parallel tangent line along the tibial cortex; the distance between these two lines is recorded as the plate–bone gap, reflecting the tightness of plate–bone apposition (Figure 5).

#### 2.4.4 Plate position

**Figure 6:**
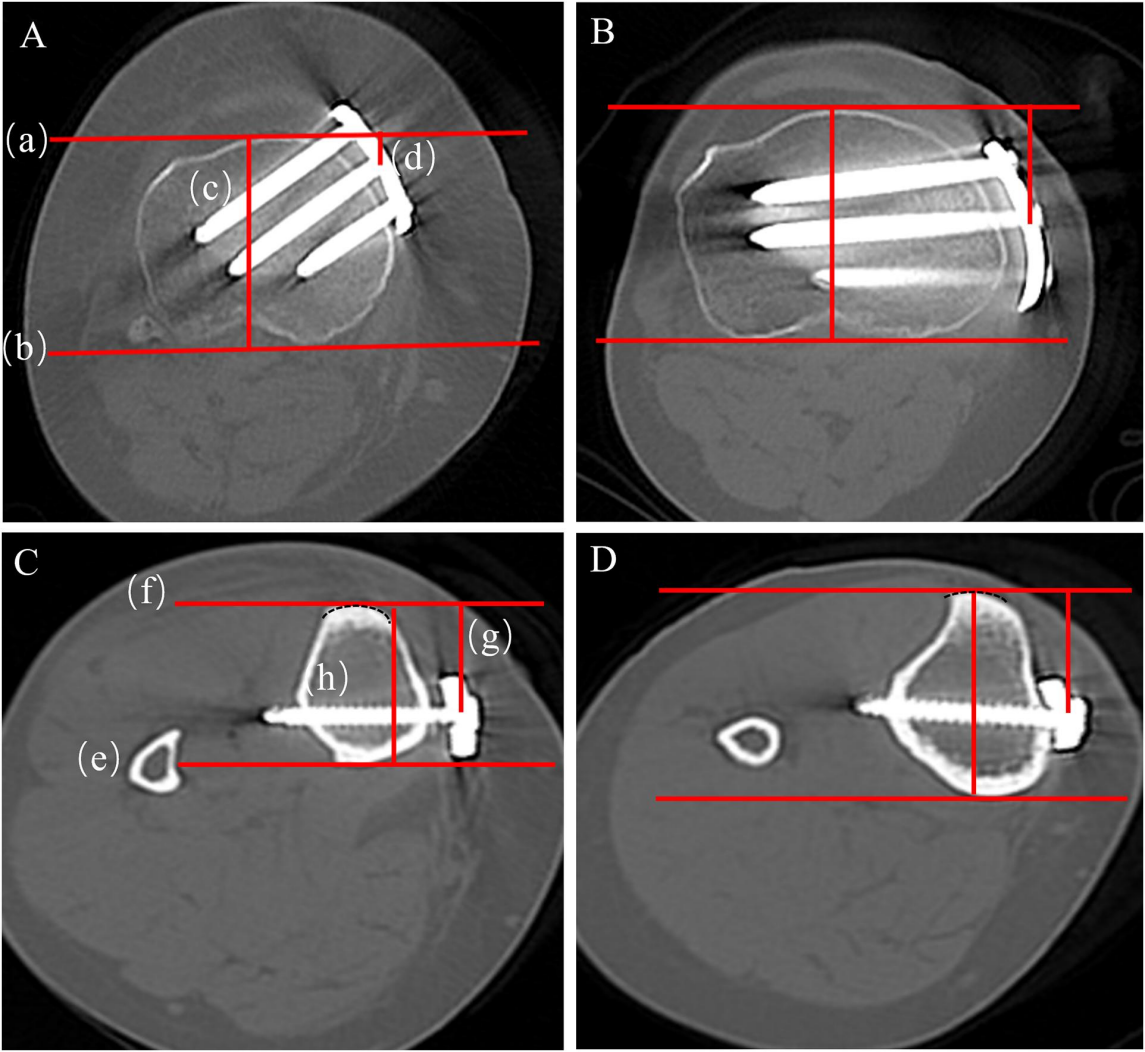
Axial CT measurement of proximal and distal plate position. **(A)** Proximal position-T-shaped locking plate; **(B)** proximal position novel locking plate; **(C)** distal position-T-shaped locking plate; **(D)** distal position novel locking plate.

Proximal plate position on axial CT is expressed as a percentile from 0 (anterior) to 100 (posterior). At the level of the proximal central hole, draw a posterior reference line (b) tangent to the posterior cortical margin of the proximal tibia, and a second line (a) parallel to (b) tangent to the anterior cortex. Measure (d), the perpendicular distance from line (a) to the centre of the plate, and (c), the distance between lines (a) and (b). where larger values indicate a more posterior placement (Figure 6).

Proximalplateposition(%)=(d/c)×100%

At the hole-1 level on axial CT, trace a semicircular curve that matches the anterior curvature of the tibial tubercle. Construct an anterior tangent line (f) that passes through the midpoint of the tubercle and is equidistant from both sides of the semicircle. Then draw a second line (e) on the posterior cortical margin parallel to line (f). Measure (g), the perpendicular distance from the anterior line (f) to the centre of the plate, and (h), the anteroposterior tibial diameter defined as the distance between the anterior (f) and posterior (e) lines (Figure 6).

Distalplateposition(%)=(g/h)×100%

Platepositionmismatch(%)=|proximalplateposition(%)−distalplateposition(%)|

Plate position mismatch (%) — higher values indicate more pronounced non-uniform torsion or displacement of the plate along the medial tibial cortex (10).

Two observers independently performed all measurements while blinded to group allocation and to each other's readings. The average of the two measurements was used for analysis. Results are reported as mean ± standard deviation (SD).

### 2.5 Statistical analysis

Data were analysed using IBM SPSS Statistics, version 22.0 (IBM Corp., Armonk, NY, USA). Normality was assessed using the Shapiro–Wilk test, and normally distributed variables are expressed as mean ± standard deviation and analyzed with Welch's *t*-test. Non-normally distributed variables are presented using quartiles and analyzed with the Mann–Whitney *U* test.

## 3 Results

### 3.1 General findings

**Figure 7:**
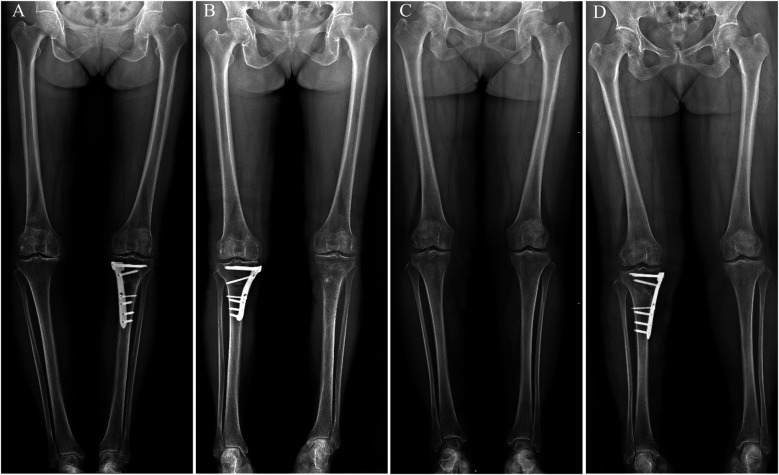
Preoperative and postoperative non–weight-bearing full-length anteroposterior radiographs of both lower limbs. **(A)** Novel locking plate-preoperative; **(B)** novel locking plate-postoperative; **(C)** T-shaped locking plate-preoperative; **(D)** T-shaped locking plate-postoperative. In both groups, the mechanical axis was corrected from preoperative varus to slight valgus or neutral.

Fourteen patients were included, and all procedures were completed uneventfully with no serious intraoperative complications. Immediate postoperative long-leg radiographs demonstrated satisfactory osteotomy reduction (Figure 7). The lower-limb mechanical axis was corrected from preoperative varus to slight valgus or neutral in all cases, and the posterior tibial slope showed no appreciable change from baseline (Table 2).

**Table 2:** Comparison of changes in radiologic parameters between the two groups.

Parameter	Novel locking plate	T-shaped locking plate
HKA (°)		
Preoperative	171.55 ± 2.82	175.20 ± 1.40
Postoperative	181.84 ± 1.51	182.85 ± 0.88
Change (Δ)	10.29 ± 1.98	7.50 ± 1.88
*P* value	<0.05	<0.05
MPTA (°)		
Preoperative	83.92 ± 2.59	86.48 ± 2.28
Postoperative	94.30 ± 0.86	92.32 ± 2.37
Change (Δ)	10.38 ± 2.63	6.41 ± 2.83
*P* value	<0.05	<0.05
PTS (°)		
Preoperative	5.14 ± 1.99	5.43 ± 1.48
Postoperative	5.02 ± 1.69	5.62 ± 1.37
Change (Δ)	−0.13 ± 0.39	0.18 ± 0.84
*P* value	0.411	0.584

Values are mean ± SD; Δ = postoperative−preoperative; *P* values are within-group (pre vs. post). HKA, hip-knee-ankle; MPTA, medial proximal tibial angle; PTS, posterior tibial slope.

### 3.2 Imaging evaluation

The results of the quantitative postoperative CT analysis are summarised in (Table 3). Significant between-group differences were observed across the five predefined metrics, including plate orientation, screw trajectory, and plate–bone apposition (*P* < 0.05).

**Table 3:** Postoperative CT comparison of plate configuration at the osteotomy site between the two groups.

Parameter	T-shaped locking plate	Novel locking plate	*P* value
Plate position in proximal fragment (%)	28.42 ± 14.40	42.27 ± 16.78	n.s.
Plate position in distal fragment (%)	66.11 ± 13.22	62.20 ± 11.20	n.s.
Mismatch of the plate position (%)	34.31 (30.57–42.08)	18.86 (14.32–23.87)	<0.05
Plate–bone gap (mm)	3.12 (2.70–3.26)	1.47 (1.35–2.28)	<0.05
A Screw length (mm)	51.89 ± 5.21	58.16 ± 7.54	n.s.
B Screw length (mm)	57.58 ± 4.80	64.38 ± 5.60	n.s.
C Screw length (mm)	38.75 ± 4.23	58.41 ± 7.98	<0.05
Screw angle (°)	27.48 ± 6.05	5.70 ± 4.80	<0.05
Plate angle (°)	62.62 ± 7.05	74.49 ± 8.76	<0.05

Data are expressed as mean ± standard deviation for normally distributed variables or median interquartile range for non-normally distributed variables. Values are presented as mean ± standard deviation. n.s. not significant.

#### 3.2.1 Plate angle

The angle between the plate and the posterior tibial condylar reference line averaged 74.49° ± 8.76° in the novel plate group, significantly greater than 62.62° ± 7.05° in the T-shaped locking plate group (*P* < 0.05).

#### 3.2.2 Screw angle and length

In the novel plate group, proximal screws ran more nearly parallel to the lateral hinge; the proximal central screw formed a significantly smaller angle with the posterior tibial condylar reference line than in the T-shaped locking plate group. The screw–plate angle of the proximal central screw was 5.70° ± 4.80° with the novel plate vs. 27.48° ± 6.05° with T-shaped locking plate (*P* < 0.05).

In the novel plate group, mean lengths were 58.16 ± 7.54 mm (screw A), 64.38 ± 5.60 mm (screw B), and 58.41 ± 7.98 mm (screw C), compared with 51.89 ± 5.21, 57.58 ± 4.80, and 38.75 ± 4.23 mm, respectively, in the T-shaped locking plate group. Screws A and B tended to be longer with the novel plate but did not reach statistical significance, whereas the difference for screw C was most pronounced and significant (*P* < 0.05).

#### 3.2.3 Plate–bone gap

The novel plate showed a mean plate–bone gap of 1.47(1.35–2.28) mm, significantly smaller than 3.12(2.70–3.26) mm with T-shaped locking plate (*P* < 0.05).

#### 3.2.4 Plate position and mismatch

Proximal plate position did not differ significantly between the novel plate and T-shaped locking plate (42.47% ± 16.78% vs. 28.42% ± 14.40%; *P* > 0.05), nor did distal position (62.20% ± 11.20% vs. 66.11% ± 13.22%; *P* > 0.05). However, plate position mismatch (|proximal−distal|) was significantly smaller with the novel plate 18.86(14.32–23.87) than with T-shaped locking plate (34.31(30.57–42.08)%; *P* < 0.05).

### 3.3 Clinical outcomes

Early postoperative clinical findings are presented in (Table 4). Both groups showed marked improvement in pain, knee function, and activity level at 2 months. The novel locking plate group achieved lower VAS scores than the conventional T-shaped plate group, indicating superior early pain relief (*P* < 0.05).

**Table 4:** Comparison of clinical outcome scores between the two groups.

Outcome	Novel locking plate	T-shaped locking plate	*P* value
VAS			
Pre-operative	6.43 ± 0.98	6.29 ± 0.49	n.s.
2-month follow-up	0.86 ± 1.07	2.43 ± 0.53	<0.05
Lysholm			
Pre-operative	29.86 ± 6.84	31.29 ± 8.48	n.s.
2-month follow-up	85.57 ± 12.90	71.86 ± 17.33	n.s.
KOOS-PS			
Pre-operative	14.71 ± 2.63	14.00 ± 2.83	n.s.
2-month follow-up	1.86 ± 2.19	4.14 ± 2.79	n.s.
Tegner			
Pre-operative	0.86 ± 0.38	1.00 ± 0.00	n.s.
2-month follow-up	4.57 ± 0.53	4.00 ± 0.00	n.s.

Values are presented as mean ± standard deviation. VAS, visual analogue scale for pain; Lysholm, Lysholm knee scoring scale; KOOS-PS, Knee injury and Osteoarthritis Outcome Score–Physical Function Shortform; Tegner, Tegner activity scale.

## 4 Discussion

This study was primarily designed as a radiographic comparison, early postoperative clinical data showed that both fixation methods effectively reduced pain and improved knee function within 2 months. The VAS score was lower in the novel locking plate group, which may be related to its better anatomic conformity and reduced anterior edge prominence and soft-tissue irritation. The lack of significant differences in other functional scores may be due to the limited sample size and short follow-up. Postoperative CT quantitative analysis likewise demonstrated that the novel plate had a smaller plate-position mismatch, better plate–bone apposition, and more favourable screw orientation, suggesting certain anatomical and biomechanical advantages.

### 4.1 Plate angle and plate position

On the axial plane, the novel locking plate sits more posteromedially. A posteriorly biased placement brings the plate closer to the tibial mechanical axis and adjacent to the dense posteromedial cortical buttress, thereby shortening the bending moment arm and providing more effective buttressing against post-osteotomy stresses. Araya et al. reported that, compared with anteromedial plating, positioning the plate more posteriorly on the medial tibia better buttresses the osteotomy gap, provides superior construct stability, and facilitates earlier weight-bearing (4). Takeuchi et al. compared anteromedial vs. more posteriorly positioned medial plating during HTO and found that the posteriorly shifted placement yielded a plate orientation closer to perpendicular to the cortex, resulting in more secure fixation (11). Moreover; excessively anterior plate placement during wedge opening may increase the PTS, adversely affecting knee stability-for example. predisposing to anterior cruciate ligament (ACL) dysfunction (12). In the present study, the posteriorly biased implantation of the novel locking plate appears to circumvent these shortcomings. The geometric structure of conventional T-shaped plates, together with the anteriorly based osteotomy corridor, limits the possibility of more posteromedial positioning. When the T-plate is shifted further posteromedially, its flat proximal T-head abuts the medial tibial crest, causing elevation of the anteromedial margin. This increases the plate–cortex distance, creates local prominence, and may compromise fixation stability. By contrast, the anatomically contoured plate used in this study has a mild curvature and a posteromedial offset, which allows more posterior/medial placement through the same surgical approach without anterior margin elevation (5, 13).

### 4.2 Screw angle and support of the mechanical axis

The proximal screw angle was significantly smaller with the novel locking plate than with T-shaped locking plate, indicating a trajectory directed more laterally rather than posteriorly. A smaller angle means the screw runs more parallel to the posterior tibial condylar line, thereby aiming closer to the lateral hinge. The lateral bony hinge is the key pivot preserved during OWHTO; the fixation construct must provide sufficient support to this region to prevent hinge fracture or excessive opening. With the conventional T-shaped locking plate, its relatively anterior placement often drives the proximal screws posteriorly toward the tibial posterior cortex, providing suboptimal support for the lateral hinge. Moreover, the screw tips lie closer to the popliteal neurovascular bundle, posing a potential risk of iatrogenic injury (14, 15). By contrast, the posteriorly conforming novel locking plate allows the proximal screws to run more nearly parallel to the posterior tibial condylar line and point laterally. This positions the screws closer to the lateral hinge to provide effective support, while keeping their tips farther from the posterior neurovascular structures (popliteal vessels and tibial nerve), thereby improving safety. Notably, the novel locking plate incorporates a purpose-designed oblique compression screw whose trajectory runs inferolaterally across the osteotomy to engage the lateral cortex of the distal fragment. Functionally, this screw “cinches” the distal segment superomedially toward the plate proximal segment, generating additional compressive force across the osteotomy and enhancing three-dimensional stability. Biomechanically, an oblique compression screw spanning the osteotomy gap increases anti-rotational stability and reduces the risk of lateral hinge fracture (13, 16). When the lateral cortical hinge fractures, the oblique screw can be applied in compression mode to re-establish stability across the hinge. When the hinge is intact, the oblique screw is used in a locking configuration, providing excellent angular stability and further reinforcing the overall construct stability of the plate system.

### 4.3 Screw length and mechanical support

Greater screw length confers multiple biomechanical advantages. First, the intraosseous length of a screw is strongly and positively correlated with its axial pull-out strength, an effect that is particularly important in cancellous bone (17, 18). In the novel plate design, posteromedial placement optimisations screw trajectories so that the A-, B-, and C-hole screws traverse a longer intraosseous path to the lateral tibial cortex. This conforming, through-trajectory configuration increases working length and cortical purchase, thereby enhancing the construct's resistance to shear and rotational loads and improving overall stability. Notably, the pronounced between-group difference in C-screw length is primarily attributable to the posteriorly biased plate position, which alters the entry point and trajectory, thereby lengthening the intraosseous path to the lateral cortex. The proximal portion of the T-shaped locking plate is relatively broad and straight, which often leads to screw trajectories deviating from the diaphyseal centre; in particular, the C-hole screw tends to point toward the posterior tibial cortex, thereby increasing the risk of popliteal artery injury and other posterior neurovascular complications. By incorporating a slight proximal posterior cant and improved anatomical conformity, the novel locking plate guides the C screw closer to the diaphyseal axis, providing a longer intraosseous working length and a more stable fixation corridor. From a clinical standpoint, optimising screw length not only increases initial construct stiffness but may also reduce postoperative implant micromotion and the risk of delayed union/nonunion. Longer screws afford greater cortical purchase and a larger bone–screw contact area, which is especially important in osteoporotic bone or when the distal metaphyseal segment is relatively thin (18). Taken together with our imaging findings, the novel locking plate-by modifying its placement and screw-hole orientation-creates a more biomechanically favourable environment for screw implantation without increasing intraoperative complexity.

### 4.4 Consistency of proximal–distal plate conformity

We used plate position mismatch to further quantify the uniformity of plate conformity along the tibial long axis between the proximal and distal levels during placement (1). This metric reflects whether the plate shows longitudinal misalignment on the tibial surface-manifesting as axial twist or anteroposterior offset. Results showed a plate position mismatch of 18.86(14.32–23.87) % with the novel locking plate, significantly lower than 34.31(30.57–42.08) % with T-shaped locking plate (*P* < 0.05), indicating markedly better proximal–distal conformity of plate placement with the novel construct. From a biomechanical perspective, a lower value indicates more consistent proximal–distal apposition of the plate to the medial tibial cortex, implying closer alignment with the anatomical axis and reducing the risk of plate twist–induced local gaps, stress shielding, and micromotion. Poor conformity can lead to: (i) aberrant screw loading with off-axis forces; (ii) discontinuous support at the plate–bone interface causing stress concentration; (iii) imprecise screw trajectories that fail to accurately target the lateral hinge; and (iv) a suboptimal healing environment, increasing the risk of nonunion or delayed union (9, 19);From a clinical perspective, plates with a large plate position mismatch may require forceful intraoperative rotation to achieve cortical conformity, which not only prolongs operative time but also increases the risk of malposition, repeated screw insertion, or re-drilling. By optimising its overall contour to conform to the medial tibial profile, the novel locking plate achieves stable apposition without additional intraoperative adjustments, thereby maintaining buttress strength while reducing subperiosteal (cortical) stripping and operative manipulation. Taken together, these results support plate position mismatch as a quantitative index of longitudinal (proximal–distal) plate conformity. Our study demonstrates a significant improvement in anatomical conformity with the novel plate, providing a methodological framework and clinical evidence to inform the design of next-generation, high-conformity internal fixation devices.

### 4.5 Plate–bone gap and fixation stability

The distance between the fixation plate and the cortical bone directly affects the stiffness of the construct. A smaller gap improves load transfer and limits micromotion, whereas a larger gap increases the bending moment and stress concentration at the interface, predisposing to loss of correction and delayed union; Conversely, when the plate is appreciably offset from the cortex, the screws behave like cantilevers, undergoing greater bending under load and thereby reducing construct stiffness and strength (20, 21). Ahmad et al.'s biomechanical testing demonstrated that positioning a locking plate 5 mm off the cortical surface significantly increased the risk of plastic deformation under cyclic loading and markedly reduced the load required to produce mechanical failure (22). Accordingly, reducing the plate–bone distance is considered critical for improving construct stability in HTO (8). Because the conventional T-shaped locking plate has a fixed geometry and is not tailored to the post-osteotomy tibial contour, partial lift-off from the cortical surface (i.e., plate–bone gapping) frequently occurs after implantation. Particularly after wedge opening, the proximal medial tibial surface becomes irregular, making full proximal plate apposition difficult; an additional cortical screw is often required to pull the plate down onto the bone surface (8). However, although an additional oblique compression screw can reduce the plate–bone gap, it may constrict the osteotomy gap and increase transverse shear stresses, thereby elevating the risk of lateral hinge fracture (16). Our measurements indicate that the novel locking plate conforms more closely to the post-correction medial tibial contour, which is expected to reduce the incidence of complications such as fixation loosening, screw breakage, and loss of correction (8, 21). In summary, the novel locking plate's superior anatomical conformity reduces the plate–bone gap and achieves secure fixation without adjunctive measures, which is crucial for enhancing initial postoperative stability after HTO.

### 4.6 Study significance and limitations

Based on early postoperative imaging data, this study provides preliminary evidence that the novel locking plate has configurational advantages in key parameters—plate–bone conformity, screw orientation, and plate position suggesting potential benefits for intraoperative placement accuracy and postoperative construct stability. This study provides clinical imaging evidence to inform the optimisation of internal fixation systems for HTO. However, this study has several limitations: the sample size is small and the follow-up is short, preventing evaluation of long-term bone union and functional recovery; moreover, biomechanical testing to validate the construct's mechanical performance is lacking. Future research should comprise prospective, multicentre studies with larger samples and long-term follow-up, complemented by *in vitro* biomechanical testing and clinical outcome evaluations, to verify whether the radiological differences observed in this study translate into superior clinical outcomes.

## 5 Conclusions

This imaging-based analysis provides preliminary evidence that, compared with the conventional T-shaped locking plate, the novel locking plate in APTT-HTO achieves superior anatomical conformity-characterised by more posteromedial plate positioning, a more favourable screw trajectory, and improved plate–bone apposition.

## Data Availability

The individual-level clinical and imaging data contain potentially identifying information and are subject to institutional and ethics restrictions. Therefore, raw data are not publicly available. De-identified datasets and measurement spreadsheets can be shared on reasonable request to the corresponding authors and with approval from the Ethics Committee of Guizhou Medical University (No. 2025140k) under a data-sharing agreement, for non-commercial research purposes only. Requests to access these datasets should be directed to Huizhong Yao, 384646369@qq.com.

## References

[1] Li OL Pritchett S Giffin JR Spouge ARI. High tibial osteotomy: an update for radiologists. Am J Roentgenol. (2022) 218(4):701–12. 10.2214/AJR.21.26659 34817194

[2] Dal Fabbro G Balboni G Di Paolo S Varchetta G Grassi A Marcheggiani Muccioli GM Lateral closing wedge high tibial osteotomy for medial knee osteoarthritis: an eleven-year mean follow-up analysis. Int Orthop. (2025) 49(7):1655–66. 10.1007/s00264-025-06525-0 40266312 PMC12179228

[3] Valcarenghi J Vittone G Mouton C Coelho Leal A Ibañez M Hoffmann A A systematic approach to managing complications after proximal tibial osteotomies of the knee. J Exp Orthop. (2023) 10(1):131. 10.1186/s40634-023-00708-7 38055158 PMC10700288

[4] Araya N Koga H Nakagawa Y Shioda M Ozeki N Kohno Y Risk factors for delayed bone union in opening-wedge high tibial osteotomy. Jt Dis Relat Surg. (2024) 35(3):546–53. 10.52312/jdrs.2024.1636 39189563 PMC11411875

[5] Park HU Bäcker HC Häner M Braun KF Petersen W. Clinical outcome after medial open-wedge high tibial osteotomy: comparison of two angular-stable locking plates—TomoFix versus LOQTEQ HTO plate. J Pers Med. (2023) 13(3):472. 10.3390/jpm13030472 36983654 PMC10053608

[6] Ryu DJ Park SJ Lee DH Kwon KB Choi GH Kim IS Does the anteromedial plate position affect proximal screw length and worsen clinical outcomes in medial open-wedge high tibial osteotomy? BMC Musculoskelet Disord. (2023) 24(1):14. 10.1186/s12891-022-06080-4 36611141 PMC9824977

[7] Shim SJ Jeong HW Park SB Lee YS. Reducing the risk of neurovascular injury with posteromedial plating and laterally directed screw insertion during opening-wedge high tibial osteotomy. Orthop J Sports Med. (2022) 10(6):23259671221098421. 10.1177/23259671221098421 35668870 PMC9163734

[8] Hayatbakhsh Z Farahmand F Karimpour M. Is a complete anatomical fit of the TomoFix plate biomechanically favourable? A parametric study using the finite element method. Arch Bone Jt Surg. (2022) 10(8):712–20. 10.22038/ABJS.2022.60928.3003 36258741 PMC9569138

[9] Wu Z Yuan D Hua D Yang L Zou Q Tian X Precise patellar tendon insertion protection and osteotomy surface advantage of transtibial tuberosity high tibial osteotomy. Orthop Surg. (2023) 15(2):639–47. 10.1111/os.13562 36419315 PMC9891937

[10] Lee ES Kim TW Jo IH Lee YS. Comparative analysis of fixation configurations and their effect on outcome after medial open-wedge high tibial osteotomy. J Orthop Sci. (2020) 25(4):627–34. 10.1016/j.jos.2019.06.018 31320145

[11] Takeuchi R Jung WH Ishikawa H Yamaguchi Y Osawa K Akamatsu Y Primary stability of different plate positions and the role of bone substitute in open-wedge high tibial osteotomy. Knee. (2017) 24(6):1299–306. 10.1016/j.knee.2017.07.015 29033262

[12] Nibe Y Takahashi T Kubo T Matsumura T Takeshita K. Effect of plate position on tibial displacement and posterior tibial slope after cyclic loading in medial open-wedge high tibial osteotomy: a biomechanical study using porcine tibia. Clin Biomech. (2023) 109:106076. 10.1016/j.clinbiomech.2023.106076 37634465

[13] Yang JC Lobenhoffer P Chang CM Chen CF Lin HC Ma HH A supplemental screw enhances biomechanical stability in medial open-wedge high tibial osteotomy. PLoS One. (2020) 15(12):e0244557. 10.1371/journal.pone.0244557 33378331 PMC7773260

[14] Lee SS Park J Lee DH. Comparison of anatomical conformity between TomoFix anatomical plate and TomoFix conventional plate in open-wedge high tibial osteotomy. Medicina. (2022) 58(8):1045. 10.3390/medicina58081045 36013511 PMC9413536

[15] Kim J Allaire R Harner CD. Vascular safety during high tibial osteotomy: a cadaveric angiographic study. Am J Sports Med. (2010) 38(4):810–5. 10.1177/0363546510363664 20200321

[16] Yang JCS Chen CF Lee OK. Benefits of opposite screw insertion technique in medial open-wedge high tibial osteotomy: a virtual biomechanical study. J Orthop Translat. (2019) 20:31–6. 10.1016/j.jot.2019.06.004 31908931 PMC6939025

[17] Fradet L Bianco RJ Tatsumi R Coleman J Aubin CÉ. Biomechanical comparison of sacral and transarticular sacroiliac screw fixation. Spine Deform. (2020) 8(5):853–62. 10.1007/s43390-020-00108-2 32274770

[18] Matsukawa K Yato Y Imabayashi H. Impact of screw diameter and length on pedicle screw fixation strength in osteoporotic vertebrae: a finite element analysis. Asian Spine J. (2021) 15(5):566–74. 10.31616/asj.2020.0353 33355846 PMC8561163

[19] Jang YW Lim D Seo H Lee MC Lee OS Lee YS. Role of an anatomically contoured plate and metal block for balanced stability between the implant and lateral hinge in open-wedge high tibial osteotomy. Arch Orthop Trauma Surg. (2018) 138(7):911–20. 10.1007/s00402-018-2918-9 29546620

[20] Evans A Glyde M Day R Hosgood G. Effect of plate–bone distance and working length on 2.0-mm locking construct stiffness and plate strain in a diaphyseal fracture gap model: a biomechanical study. Vet Comp Orthop Traumatol. (2024) 37(1):1–7. 10.1055/s-0043-1771198 37473771

[21] Hayatbakhsh Z Farahmand F. Effects of plate contouring quality on the biomechanical performance of high tibial osteotomy fixation: a parametric finite element study. Proc Inst Mech Eng H. (2022) 236(3):356–66. 10.1177/09544119211069207 35001727

[22] Ahmad M Nanda R Bajwa AS Candal-Couto J Green S Hui AC. Biomechanical testing of the locking compression plate: when does the distance between bone and implant significantly reduce construct stability? Injury. (2007) 38(3):358–64. 10.1016/j.injury.2006.08.058 17296199

[23] Yildirim K Beyzadeoglu T. Removal rate of the TomoFix system after high tibial osteotomy is higher than reported. Rev Bras Ortop. (2022) 58(2):326–30. 10.1055/s-0042-1750836 PMC10212620 37252299

